# The impact of health policies and the COVID-19 pandemic on exclusive breastfeeding in Chile during 2009–2020

**DOI:** 10.1038/s41598-023-37675-z

**Published:** 2023-07-01

**Authors:** Deborah Navarro-Rosenblatt, Tarik Benmarhnia, Paula Bedregal, Sandra Lopez-Arana, Lorena Rodriguez-Osiac, Maria Luisa Garmendia

**Affiliations:** 1 PhD Program, School of Public Health, University of Chile, Av. Independencia 939, Independencia, Santiago Chile; 2 Department of Family Medicine and Public Health, University of California at San Diego, California, 9500 Gilman Drive, La Jolla, CA 92093 USA; 3 School of Public Health, Pontifical Catholic University of Chile, Av. Libertador Bernardo O’Higgins 340, Santiago, Chile; 4 Department of Nutrition, Faculty of Medicine, University of Chile, Av. Independencia 1027, Santiago, Chile; 5 School of Public Health, University of Chile, Av. Independencia 939, Santiago, Chile; 6 Institute of Nutrition and Food Technology, University of Chile, Av. El Libano 5524, Macul, Santiago, Chile

**Keywords:** Epidemiology, Health policy, Public health, Nutrition

## Abstract

In 2011, Chile added 12 mandatory extra weeks of maternity leave (ML). In January 2015, a pay-for-performance (P4P) strategy was included in the primary healthcare system, incorporating exclusive breastfeeding (EBF) promotion actions. The COVID-19 pandemic led to healthcare access difficulties and augmented household workloads. Our aim was to evaluate the effect of a 24-week ML, the P4P strategy, and COVID-19 on EBF prevalence, at 3 and 6 months in Chile. Aggregated EBF prevalence data from public healthcare users nationwide (80% of the Chilean population) was collected by month. Interrupted time series analyses were used to quantify changes in EBF trends from 2009 to 2020. The heterogeneity of EBF changes was assessed by urban/setting and across geographic settings. We found no effect of ML on EBF; the P4P strategy increased EBF at 3 months by 3.1% and 5.7% at 6 months. COVID-19 reduced EBF at 3 months by  − 4.5%. Geographical heterogeneity in the impact of the two policies and COVID-19 on EBF was identified. The null effect of ML on EBF in the public healthcare system could be explained by low access from public healthcare users to ML (20% had access to ML) and by an insufficient ML duration (five and a half months). The negative impact of COVID-19 on EBF should alert policy makers about the crisis's effect on health promotion activities.

## Introduction

Exclusive breastfeeding (EBF) is known to be more than a source of nutrition for children. It is also a foundation for the growth and development of the immune, gastric, and cognitive systems and is a key factor for preventing chronic diseases in adulthood^1–3^. There are also various documented benefits of EBF for mothers, including the prevention of breast and ovarian cancer, other chronic diseases, and postpartum depression^1^. In 1990, the World Health Organization (WHO) issued the Innocenti Declaration on the Protection of Breastfeeding^4^, highlighting the significance of EBF and the importance of having EBF for at least 6 months. The WHO has set a global target for EBF at 6 months of at least 50% by 2025 and 70% by 2030^5^. However, few countries have achieved this goal, with a global EBF rate of only 38% at 6 months in 2018^6^.

Several strategies have succeeded in promoting EBF, including paid maternity leave (ML)^1,7^. Evidence has reported longer EBF with extended duration of ML; women with ML of at least three months were three times more likely to continue EBF than women who returned to work earlier^8^. Pay-for-performance (P4P) strategies in primary healthcare have also been successful in improving health outcomes. P4P, described as financial incentives or rewards for healthcare workers, aims to help achieve primary healthcare centers (PHCC) health outcomes^9,10^. However, the potential impact of P4P strategies on EBF has not yet been investigated.

In 2011, Chile extended the mandatory fully-paid existing 12 weeks of ML by 12 extra^11^, bringing the total to 24 weeks. The ML in Chile allows women working with formal contracts and freelancer contracts with up-to-date pension scheme payments to access fully paid ML for five and a half months (24 weeks). A descriptive study showed a 1.8% increase in EBF prevalence at 6 months after the implementation of the extended ML^12^. Moreover, a recent study reported no effect of the extended ML on EBF by socio-economic status^13^. In addition, the last Chilean breastfeeding survey reported a prevalence of EBF of 56%; however, no national or regional analysis of the following trend has been published^14^.

Access to extra 12 weeks of ML could have increased EBF prevalence at 3 and 6 months. However, no studies have quantified this effect. In January 2015, Chile added EBF promotion to its P4P strategies in PHCCs^15^, aiming to strengthen efforts to promote EBF at 6 months. The P4P strategy included a bonus payment three times a year on top of PHCCs’ health professionals’ regular salary who met the EBF at 6 months’ target (details of the P4P strategy in Table 1).

**Table 1:** Details of the three-time events included in the interrupted time series analyses.

Extended maternity leave^1,2^	In October 2011, 12 extra weeks were added to the previous 12 weeks of mandatory and fully paid maternity leave (ML), totaling 24 weeks (five and a half months). More flexible norms were also incorporated, allowing women with informal jobs to access ML. Nonetheless, women with unofficial jobs required health insurance payments of at least 6 months during the 12 months before pregnancy and pension scheme outflows of a minimum of 6 months prior to the 12 months before pregnancy. Chile has been reported to have a high prevalence of informal workers, which could affect access to work benefits, like the paid ML2
Pay-for-performance (P4P) strategy to promote EBF at 6 months in primary healthcare^3^	The pay-for-performance (P4P) strategy included in this study aims to promote exclusive breastfeeding (EBF) at 6-months in the primary healthcare sector. The P4P strategy is defined as a paid incentive to primary healthcare professionals for achieving healthcare goals previously decreed by law. The P4P strategy includes individual and group counselling with a midwife or nutritionist, specialised training for health professionals in primary healthcare, specialised counselling for mothers and their families
COVID-19 pandemic in Chile, up to November 2020	On 15th March all educational institutions were shut until November 2020 and remote work was pushed. From the 22nd of March 2020, the whole country was under curfew from 9 pm to 5 am. In May and June 2020, most of the northern and central regions were in lockdown. Southern regions had fewer locations under lockdown^4^. From April to November 2020, the public healthcare centres focused their activities on COVID-19 case traceability, cancelling the majority of the health check-ups, and only returning to previous standards in early 2022

^1^Gobierno de Chile. Ley 20,545 Modifica las Normas de Protección a la Maternidad e Incorpora el Permiso Postnatal Parental [InternetV]. Chile: Gobierno de Chile; 2011. Available from: https://www.leychile.cl/Navegar?idNorma=1030936.

^2^INE. Encuesta Nacional de Empleo [Internet]. Chile: Instituto Nacional de Estadísticas; 2018 [cited 2019 Feb 16]. Available from: https://www.ine.cl/estadisticas/laborales/ene?categoria=Situaci%C3%B3n%20de%20Fuerza%20de%20Trabajo.

^3^Biblioteca del Congreso Nacional. Resolución Exenta 880 [Internet]. Chile: Gobierno de Chile. 2014. Available from: https://www.leychile.cl/N?i=1067767&f=2014-10-02&p=

4Lockdown during the COVID-19 pandemic in Chile. https://es.wikipedia.org/wiki/Confinamiento_por_la_pandemia_de_COVID-19_en_Chile, 2020–2022.

The COVID-19 pandemic led to difficulties in accessing healthcare services, increased women’s household workload, and further impoverished Chile’s population, particularly women, which probably affected EBF practices. In addition, the COVID-19 pandemic represented a reduction of healthcare access in the public healthcare system^16,17^.

Chile is a long, thin country, with notable differences in access to healthcare in rural, isolated, and more deprived locations^18^, with fewer health checkups access and less medical support in rural areas compared to urban, central, and more privileged locations^19^. This might explain the previously reported variability in EBF prevalence between Chilean regions, with lower rates in the northernmost and southernmost regions^12^.

The aim of this study is to evaluate the impact of the mandatory extended ML in 2011, the P4P strategy in 2015, and the COVID-19 pandemic in Chile in the prevalence of EBF at 3 and 6 months, measured using national data. In addition, this work explores the possible effect of these three time-events on EBF stratified by urban and rural areas and by geographical location.

## Methods

### Study design and setting

We performed interrupted time-series analyses (ITSA^20^) to quantify the changes in EBF prevalence, following the implementation of two policies and the COVID-19 pandemic. We measured EBF with aggregated municipal (municipality is the smallest administrative unit in Chile) data. Administrative permissions were required to use and access the primary healthcare system national database employed in this study.

In brief, an ITSA assesses whether there are modifications over time in the trends of a specific outcome (in this case, EBF), after the introduction of an intervention and compares them to an estimated counterfactual trend based on pre-treatment observations (historical control group)^21^.

This is a nationwide study, with data from the whole public healthcare system service users, which compromises approximately 80% of the total Chilean population^22^. The other 20% are attended in the private health system, which is entirely based on private insurers^23^.

### Variables and data collection

#### Municipal exclusive breastfeeding percentage

EBF (outcome) included monthly records from 345 Chilean municipalities. Data were obtained from public healthcare system feeding registries recorded during health checkups by health professionals at 3 and 6 months. Health professionals registered if a child had EBF, partial BF, or formula, or solid food, following the WHO definition of EBF: “Exclusive breastfeeding refers to being uniquely fed by breastmilk, from the mother, wet nurse, or pumped milk, without receiving any other kind of food or liquid unless a health professional prescribes a medicament, such as syrup or drops, vitamins or minerals”^24^. This definition includes children who occasionally receive a small quantity of water. The data of children with up-to-date checkups were collected in the same manner as the EBF data. The following definition was used for up-to-date checkups: “children who attended health checkups performed at the age of one, three, six, 12, and 24 months^24^”. Using the category “children fed with EBF”, the EBF percentage was calculated using the monthly absolute numbers of children with EBF at 3 and 6 months of each PHCC and the absolute number of children with health checkups from the same age group and location.

Each PHCC is responsible for sending monthly aggregated data to the Regional Health Office, where the information is consolidated by municipality (each municipality has more than one PHCC), revised, and sent to the Health Ministry’s Department of Statistics and Health Information.

We included the EBF cut-off of 3 months because, in Chile, the previous 12-week ML corresponded to two and a half months; hence EBF at 3 months could provide a comparison before and after the extension of the ML. In addition, EBF at 6 months was assessed because this corresponds to the WHO exclusive breastfeeding recommendation^4,25^.

#### Interventions

This study included three time-events:

- October 2011: implementation of 12 additional weeks of fully paid mandatory ML, bringing the total to 24 weeks. The introduction of the extended ML also incorporated more flexible terms to access ML, aiming to increase ML access in women with informal jobs. The preintervention period was from January 2009 to September 2011, and the intervention period was from October 2011 to December 2014.
- January 2015: The P4P strategy was implemented to encourage health professionals to increase the promotion and support of EBF to achieve the 6-month EBF national goal. The preintervention period was from January 2009 to December, and the intervention period was from January 2015 to November 2020.
- March 2020: The beginning of the COVID-19 pandemic in Chile. COVID-19 represented a reduction in healthcare services, including EBF promotion. EBF data were available until November 2020. The preintervention period was from January 2015 to March 2020, and the intervention period was from April 2020 to November 2020.

#### Covariables

##### Urban–rural areas

Rural municipalities were defined according to the parameters used by the 2017 Chilean Census: “a rural entity is a human settlement with a population of 1000 or less, or 1001 and 2000, where more than 50% have jobs in the primary sector (farming, fishing, agriculture, and similar), or where there is a small settlement that meets the population criteria to be defined as urban, but not the requirements of amassing, continuity or concentration of constructions”. In addition, we also considered rural a municipality that had more than 50% of its population living in rural areas. Thirty percent of the municipalities (106 municipalities) were classified as rural (approximately 3.5 million people)^26^.

##### Geographic macrozones

**Figure 1:**
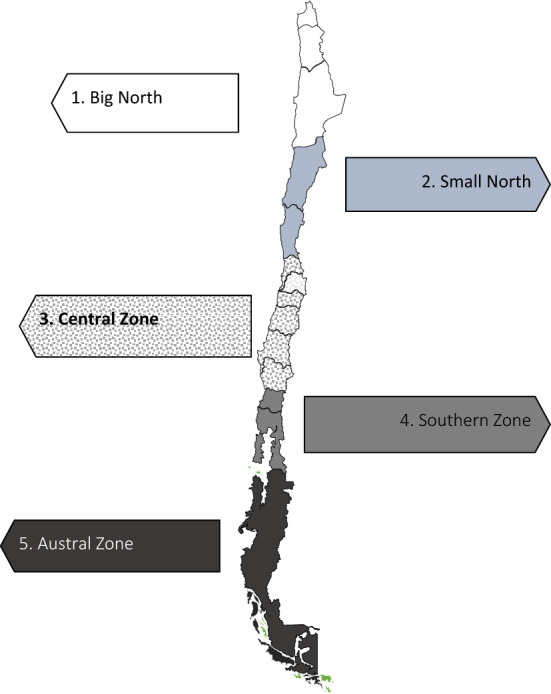
Map of Chilean topographical zones.

Municipalities were aggregated into five (topographic) macrozones, which are defined by morphology, geography, natural resources, climate, and the type of work their inhabitants perform: Big North, Small North, Central, Southern, and Austral (Fig. 1)^27^. These five macrozones are known as the economic and geographical macrozones. For example, the population in the Northern macrozone works mainly in mining and live in isolated desertic areas, whereas, in the Austral zone, the main economic development is related to agriculture and exports^28^.

#### Other covariables included in the analyses

##### Seasonality

Seasonality was included in the models as a fixed-effect monthly dummy^1–12^, with the first measure (January) serving as the reference category. Seasonal dummies help to capture possible seasonal effects.

##### ML access

The ML monthly access was calculated as a national percentage using the absolute number of ML issued in the Public Health System (Maternity Leave Management and Information System [SIMAT] from the Social Welfare Superintendence, SUSESO)^29^ and the number of newborns in the public health system. The average ML access rate across the whole study period was 20% (ranging from 14 to 24%), with no major increase or decrease after the implementation of the extra 12 weeks of ML. These results on ML access agreed with a previous study that reported similar trends and low access of ML in women accessing the public healthcare system^30^. Although the ML is mandatory by law for all mothers, only mothers with a formal contract or freelancer contracts with pension scheme up-to-date payments can access the 24-week ML.

### Data analysis

The following regression model was specified to estimate the change in municipal EBF prevalence after each of the three-time events:

1 $$EBFt(Y_{t,i} ) = \beta_{0} + \beta_{1} T_{ti} + \beta_{2} X_{ti} + \beta_{3} X_{ti} T_{ti} + \beta_{4} + e_{ti}$$

*EBF* represents the outcome at time point (months) *T* for each equally spaced time point *t* at each individual level _*i*.._ *T*_*ti*_ indicates the time since the beginning of the study (up to 143 months). *X*_*ti*_ represents an intervention dummy variable (preintervention period 0, intervention 1), and *X*_*ti*_*T*_*ti*_ is an interaction term. *β*_*0*_ is the intercept and EBF prevalence at the beginning of the study period. *β*_*1*_ represents the outcome trend before the intervention, which helps to quantify the trend in the absence of the intervention.* β*_*2*_ is the slope change in the outcome following the intervention. *β*_*3*_ estimates the change in the EBF trend in the long term after the intervention, comparing it with the EBF trend before the intervention. ${\beta }_{4}$(months 1–12) represents the seasonality adjustment as a seasonal dummy. *E*_*ti*_ represents the errors in the model over time. In this study, the time series dataset had three breakpoints (extended ML, P4P strategy, and COVID-19 pandemic), each analysed using the above model in independent segments to avoid an overlapping effect^31^. All the above-mentioned coefficients are shown in the results tables, but the results and discussion will refer only to the slope change coefficient (*β*_*2*_*).*

The ITSA was conducted using the “XTITSA module” in Stata Software (v.17), considering each municipality as individual-level data^20,32^. Autocorrelation was evaluated using the Durbin-Watson test*.* As small evidence of autocorrelation was identified, analyses were performed with correction for autocorrelation. The Cochran Q test for heterogeneity was used to analyse possible statistical differences between strata of urban and rural areas and in the five geographic macrozones, using the national results as reference for the urban and rural analyses and the Central Zone as reference for the macrozone analyses^33^.

### Ethics

This study was approved by the Ethics in Human Research Committee (*Comité de Ética de Investigación en Seres Humanos*) of the Faculty of Medicine of the University of Chile, under the Project: No 069-2021 and file number N° 045. Informed consent was not required because the databases used in this study were anonymised and the records were aggregated. Our study did not involve human or animal laboratory experiments.

## Results

**Figure 2:**
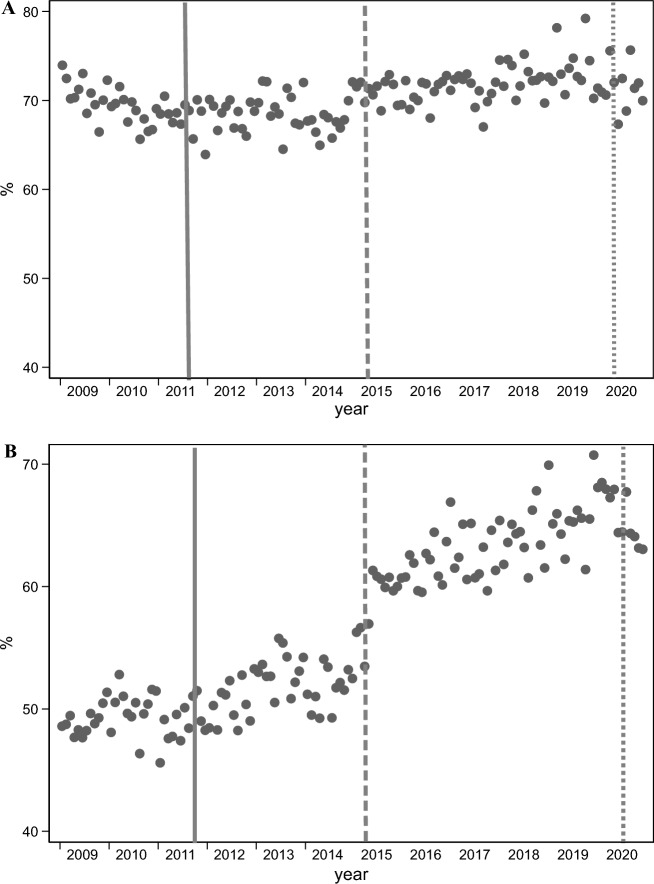
(**A**) Exclusive breastfeeding at the 3-month trend, from January 2009 to November 2020. (**B**) Exclusive breastfeeding at the 6-month trend, from January 2009 to November 2020. Legend: Dark line: Extended maternity leave; big dash line: Pay for performance strategy; small dash line: COVID-19.

Figure 2A,B show the EBF prevalence trend at 3 and 6 months, respectively. Figure 2A shows that the EBF trend at 3 months is stable, whereas the EBF trend at 6 months changes sharply during the study period. Regarding EBF prevalence, the mean nationwide EBF percentage at 3 months went from 69.5% before the implementation of the extended ML (October 2011) to 71.2% after the beginning of the COVID-19 pandemic (March 2020). The EBF prevalence at 6 months was 49.2% before the extended ML and 64.6% when COVID-19 started (Table 2). The prevalence for 3 and 6 months in urban settings was higher throughout the study period compared to rural settings. The EBF prevalence trend by urban and rural settings and geographical zone are shown in Table 2.

**Table 2:** Exclusive breastfeeding mean prevalence (95% CI), at 3 and 6 months, according to public policies and COVID-19, by urban/rural and macrozones, Chile 2009–2020.

		Extended maternity leave		Primary healthcare strategy		COVID-19	
		Before*	After**	Before*	After**	Before*	After**
3 months	Total country	69.5 (69.0, 70.1)	68.4 (67.9, 69.0)	69.0 (68.6, 69.4)	71.6 (71.2, 72.1)	71.6 (71.2, 72.1)	71.2 (69.3, 73.1)
	Urban	68.1 (67.5, 68.7)	66.8 (66.3, 67.4)	67.5 (67.1, 67.9)	70.2 (69.8, 70.7)	70.2 (69.8, 70.7)	70.5 (68.3, 72.6)
	Rural	74.5 (73.1, 75.9)	74.8 (73.4, 76.3)	74.7 (73.6, 75.7)	78.2 (76.8, 79.7)	78.7 (77.2, 80.3)	73.5 (69.8, 77.3)
	Big north zone	71.2 (67.9, 74.6)	67.0 (63.6, 70.3)	69.2 (66.9, 71.6)	69.5 (67.7, 71.4)	70.2 (68.3, 72.1)	63.8 (57.2, 70.5)
	Small north zone	66.1 (64.1, 68.1)	64.1 (61.6, 66.5)	65.3 (63.7, 66.8)	71.1 (69.1, 73.1)	70.3 (68.2, 72.4)	82.7 (75.7, 89.6)
	Central zone	68.0 (67.4, 68.6)	67.7 (67.2, 68.3)	67.9 (67.4, 68.3)	71.4 (70.9, 71.9)	71.4 (70.9, 71.9)	71.2 (68.9, 73.6)
	Southern zone	76.4 (75.0, 77.8)	74.9 (73.0, 76.7)	75.9 (74.7, 77.0)	75.4 (73,8, 77.5)	75.9 (73.9, 77.9)	73.0 (67.7, 78.2)
	Austral zone	72.0 (67.6, 76.3)	75.4 (71.7, 79.1)	73.6 (70.8, 76.5)	70.0 (67.0, 73.0)	69.9 (66.6, 73.1)	70.8 (64.0, 77.6)
6 months	Total Country	49.2 (48.6, 49.8)	51.5 (50.9, 52.1)	50.3 (49.9, 50.8)	62.4 (61.9, 62.9)	62.2 (61.7, 62.7)	64.6 (62.5, 66.7)
	Urban	47.1 (46.5, 47.8)	48.9 (48.6, 49.5)	48.0 (47.6, 48.5)	60.3 (59.8, 60.8)	60.2 (59.7, 60.7)	61.9 (59.6, 64.1)
	Rural	56.1 (54.5, 57.7)	61.4 (59.8, 63.0)	58.6 (57.5, 59.8)	71.4 (70.1, 72.7)	71.1 (69.7, 72.5)	74.6 (70.0, 79.2)
	Big north zone	54.3 (50.7, 57.9)	55.7 (52.0, 59.4)	54.9 (52.4, 57.5)	59.9 (57.4, 62.3)	60.4 (57.9, 62.9)	52.9 (42.4, 63.4)
	Small north zone	43.3 (41.2, 45.5)	48.2 (45.2, 51.1)	45.4 (43.6, 47.1)	61.1 (59.0, 63.1)	60.6 (58.6, 62.7)	66.4 (56.8, 76.0)
	Central zone	46.4 (45.7, 47.1)	49.8 (49.1, 50.4)	48.2 (47.7, 48.7)	61.6 (61.1, 62.2)	61.4 (60.9, 62.9)	64.2 (61.6, 66.8)
	Southern zone	56.0 (54.8, 57.9)	59.0 (57.1, 60.9)	57.4 (56.2, 58.7)	67.7 (66.1, 69.3)	67.6 (65.9, 69.3)	69.0 (63.7, 74.4)
	Austral zone	60.8 (56.0, 65.6)	62.6 (58.6, 66.7)	61.7 (48.6, 49.6)	64.9 (61.2, 68.6)	64.6 (61.0, 68.3)	63.5 (51.8, 75.2)

*Before: 1. Extended maternity leave: January 2009 to September 2011; 2. Primary Healthcare Strategy: January 2009 to December 2014; 3. COVID-19: January 2015/March 2020.

**After: 1. Extended maternity leave: October 2011 to December 2014; 2. Primary Healthcare Strategy: January 2015 to November 2020; 3. COVID-19: April 2020–November 2020.

Table 3 presents the adjusted estimates of the ITSA for municipal EBF prevalence at 3 and 6 months after each of the three-time events. The nationwide results reported no effect of the extended ML on EBF at 3 or 6 months, whereas the P4P strategy reported a positive effect (3.1% at three and 5.7% at 6 months), and COVID-19 decreased EBF only at 3 months by 4%.

**Table 3:** Exclusive breastfeeding trend at 3 and 6 months, after the implementation of the extended maternity leave (ML), the pay-for-performance (P4P) strategy, and the COVID-19 pandemic. Chile 2009–2020.

Outcome	%EBF change (95% CI)	
	3 months	6 months
Extended maternity leave, 2011		
EBF** slope change after ML^†^ (β_2_)	1.23 (− 0.18, 2.65)	0.30 (− 1.19, 1.81)
Change in EBF trend from Nov.2011–Dec.2014 relative to Jan.2009–Oct.2011 (β_3_)	0.11 (0.05, 0.18)	0.03 (− 0.03, 0.10)
Post-intervention linear trend (monthly change from Nov.2011—Dec.2014)	0.01 (− 0.03, 0.05)	0.08 (0.03, 0.12)
Primary healthcare strategy, 2015		
EBF slope change after P4P^‡^ (β_2_)	3.10 (2.03, 4.18)	5.69 (4.61, 6.78)
Change in EBF trend from Jan.2009–Dec. 2014 relative to Jan.2015–Nov.2020 (β_3_)	0.07 (0.05, 0.10)	0.08 (0.05, 0.11)
Post-intervention linear trend (monthly change from Jan.2015–Nov.2020)	0.05 (0.03, 0.07)	0.15 (0.12, 0.17)
COVID-19 Pandemic, 2020		
EBF slope change after COVID-19 (β_2_)	− 4.51 (− 7.93, − 1.09)	− 2.16 (− 5.52, 1.19)
Change in EBF trend from Jan. 2015–Mar.2020 relative to Apr. 2020–Nov.2020 (β_3_)	0.21 (− 0.52, 0.94)	− 0.85 (− 1.56, − 0.13)
Post-intervention linear trend (monthly change from Apr.2020–Nov.2020)	0.28 (− 0.45, 1.02)	− 0.67 (− 1.39, 0.03)

Adjusted for seasonality.

**EBF exclusive breastfeeding.

^†^*ML* extended maternity leave.

^‡^*P4P* primary healthcare strategy.

Stratified analyses by urban and rural areas (Table 4) at 3 months reported no impact of the extended ML, nor COVID-19, in both urban and rural settings, with no heterogeneity within subgroups for ML and COVID-19. The EBF prevalence at 3 months increased in both urban and rural locations after the implementation of the P4P strategy, with no heterogeneity. The extended ML also did not impact EBF at 6 months when stratified by urban and rural settings. On the other hand, the P4P showed an increase in EBF at 6 months only in urban areas, with a 6.46% (95% CI 5.34, 7.57) increase and with heterogeneity (p-value 0.012). Regarding geographical macrozones, stratified analyses showed a positive impact of the extended ML on EBF prevalence at 3 months in two macrozones: Small North 6.35% (95% CI 1.34, 11.35) and the Austral Zone 9.28% (95% CI 2.96, 15.61), with heterogeneity observed in all strata, except in the Southern Zone. For EBF at 6 months, no impact of the 24 weeks ML was observed, but heterogeneity was identified in the Big North and Southern Zone. The P4P strategy showed an increase in EBF at 3 months in all macrozones besides the Austral Zone, with heterogeneity in that zone. At 6 months, the P4P reported an increase in EBF in all macrozones. The COVID-19 pandemic decreased the EBF prevalence at 3 months in the Big North by 19.51% (95% CI − 28.55, − 10.47), with heterogeneity only in that zone. No changes in EBF at 6 months were observed during COVID-19 (Table 5).

**Table 4:** Exclusive breastfeeding (EBF) trend at 3 and 6 months, after the implementation of the extended maternity leave, pay-for-performance (P4P) strategy and the COVID-19 pandemic in urban and rural areas. Chile 2009–2020.

Outcome	%EBF change (95% CI)		%EBF change (95% CI)	
	3 months		6 months	
	Extended maternity leave, 2011			
	Urban	Rural	Urban	rural
	P Heterogeneity^#^ = 0.696		P Heterogeneity = 0.867	
EBF slope change after ML (β_2_)	1.37 (− 0.09, 2.83)	0.56 (− 3.27, 4.40)	0.30 (− 1.22, 1.83)	0.24 (− 3.77, 4.25)
Change in EBF trend from Nov.2011–Dec.2014 relative to Jan.2009–Oct.2011(β_3_)	0.14 (0.07, 0.21)	0.00 (− 0.16, 0.18)	0.09 (0.02, 0.16)	− 0.16 (− 0.35, 0.17)
Post-intervention linear trend (monthly change from Nov.2011–Dec.2014)	0.02 (− 0.02, 0.06)	− 0.01 (− 0.14, 0.11)	0.09 (0.04, 0.14)	0.03 (− 0.09, 0.15)

	Primary healthcare strategy, 2015			
	Urban	Rural	Urban	Rural
	P Heterogeneity = 0.258		P Heterogeneity = 0.012	
EBF slope change after P4P (β_2_)	2.71 (1.62, 3.81)	4.54 (1.38, 7.71)	6.46 (5.34, 7.57)	2.63 (− 0.30, 5.56)
Change in EBF trend from Jan.2009 to Dec.2014 relative to Jan. 2015–Nov.2020(β_3_)	0.09 (0.06, 0.12)	− 0.01 (− 0.09, 0.06)	0.09 (0.06, 0.12)	0.03 (− 0.04, 0.11)
Post-intervention linear trend (monthly change from Jan.2015 to Nov.2020)	0.07 (0.04, 0.09)	− 0.02 (− 0.09, 0.03)	0.15 (0.12, 0.17)	0.15 (0.08, 0.21)

	COVID-19 Pandemic, 2020			
	Urban	Rural	Urban	Rural
	P Heterogeneity = 0.311		P Heterogeneity = 0.348	
EBF slope change after COVID-19 (β_2_)	− 3.55 (− 7.12, 0.9)	− 8.66 (− 18.1, 0.76)	− 1.42 (− 4.92, 2.06)	− 6.14 (− 15.4, 3.16)
Change in EBF trend from Jan.2015–Mar.2020 relative to Apr. 2020–Nov.2020(β_3_)	0.137 (− 0.64, 0.90)	0.50 (− 1.57, 2.58)	− 1.01 (− 1.75, − 0.27)	− 0.00 (− 2.04, 2.04)
Post-intervention linear trend (monthly change from Apr.2020 to Nov.2020)	0.21 (− 0.55, 0.98)	0.51 (− 1.56, 2.59)	− 0.83 (− 1.57, − 0.09)	0.17 (− 1.86, 2.21)

*Adjusted for seasonality.

**EBF exclusive breastfeeding.

^†^*ML* extended maternity leave.

^‡^*P4P* primary healthcare strategy.

^#^P-heterogeneity for EBF change after the event coefficient.

**Table 5:** Exclusive breastfeeding trend at 3 and 6 months after the implementation of the extended maternity leave (ML), the pay-for-performance (P4P) strategy, and the COVID-19 pandemic by macrozones. Chile 2009–2020.

Intervention	%EBF change (95% CI)				
	3 months				
	Big North	Small North	Central	Southern	Austral
Extended maternity leave					
EBF slope change after ML (β_2_)	5.66 (− 0.25, 11.5)	6.35 (1.34, 11.35)	0.76 (− 0.76, 2.29)	2.80 (− 1.20, 6.80)	9.28 (2.96, 15.61)
P Heterogeneity*	0.001	< 0.001	Reference	0.161	< 0.001
EBF trend change^†^ (β_3_)	0.03 (− 0.23, 0.31)	0.47 (0.24, 0.70)	0.13 (0.6, 0.20)	0.23 (0.05, 0.41)	0.06 (− 0.22, 0.35)
Post-intervention linear trend^‡^	− 0.10 (− 0.30, 0.08)	0.39 (− 0.12, 0.20)	0.02 (− 0.01, 0.07)	0.02 (− 0.11, 0.15)	− 0.14 (− 0.34, 0.06)
Primary healthcare strategy					
EBF slope change after P4P (β_2_)	9.81 (5.89, 13.8)	5.73 (1.90, 9.55)	3.93 (2.78, 5.09)	3.54 (0.15, 6.92)	0.26 (− 4.33, 4.87)
P Heterogeneity*	< 0.001	0.112	Reference	0.727	0.001
EBF trend change^§^ (β_3_)	0.02 (− 0.07, 0.11)	0.20 (0.10, 0.30)	0.06 (0.03, 0.09)	0.08 (− 0.00, 0.16)	0.15 (0.32, 0.26)
Post-intervention linear trend^¶^	− 0.00 − (0.07, 0.07)	0.14 (0.06, 0.22)	0.03 (0.01, 0.06)	0.02 (− 0.04, 0.09)	0.14 (0.05, 0.24)
COVID-19					
EBF slope change after COVID-19 (β_2_)	− 19.5 (− 28.5, − 10.4)	− 7.38 (− 20.5, 5.76)	− 3.61 (− 7.45, 0.23)	− 8.48 (− 18.6, 1.73)	− 7.77 (− 21.4, 5.86)
P Heterogeneity*	< 0.001	0.316	Reference	0.184	0.27
EBF trend change^#^ (β_3_)	2.21 (0.32, 4.10)	1.01 (− 1.83, 3.86)	− 0.00 (− 0.82, 0.82)	0.71 (− 1.53, 2.96)	− 1.41 (− 4.42, 1.59)
Post-intervention linear trend**	2.27 (0.37, 4.16)	1.14 (− 1.69, 3.99)	0.05 (− 0.77, 0.87)	0.80 (− 1.44, 3.05)	− 1.19 (− 4.20, 1.81)
6 months					
Extended maternity leave					
EBF slope change after ML (β_2_)	− 1.80 (− 7.61, 4.00)	− 4.47 (− 9.73, 0.79)	0.47 (− 1.12, 2.07)	− 0.30 (− 4.36, 3.75)	− 11.6 (− 23.9, 0.79)
P Heterogeneity*	0.149	0.001	Reference	0.612	< 0.001
EBF trend change ^†^ (β_3_)	0.09 (− 0.17, 0.36)	0.47 (0.23, 0.71)	0.07 (0.00, 0.15)	0.11 (− 0.05, 0.29)	− 0.38(− 0.93, 0.16)
Post-intervention linear trend^‡^	0.08 (− 0.09, 0.26)	0.38 (0.22, 0.54)	0.08 (0.03, 0.13)	0.09 (− 0.03, 0.22)	0.20 (− 0.14, 0.54)
Primary healthcare strategy					
EBF slope change after P4P (β_2_)	12.6 (8.85, 16.3)	6.54 (2.84, 10.2)	7.20 (6.03, 8.36)	4.40 (1.48, 7.32)	4.84 (0.39, 9.29)
P Heterogeneity*	< 0.001	0.558	Reference	0.01	0.039
EBF trend change^§^ (β_3_)	0.01 (− 0.08, 0.10)	0.15 (0.05, 0.24)	0.07 (0.04, 0.10)	0.14 (0.06, 0.21)	0.14 (0.02, 0.25)
Post-intervention linear trend^¶^	0.01 (− 0.05, 0.09)	0.21 (0.14, 0.29)	0.14 (0.11, 0.16)	0.16 (0.10, 0.22)	0.17 (0.08, 0.27)
COVID-19, 2020					
EBF slope change after COVID-19 (β_2_)	1.90 (− 6.73, 10.5)	− 2.66 (− 12.5, 7.25)	0.99 (− 2.72, 4.72)	− 2.04 (− 10.3, 6.26)	7.57 (− 4.73, 19.8)
P Heterogeneity*	0.794	0.305	Reference	0.382	0.07
EBF trend change^#^ (β_3_)	− 0.31 (− 2.20, 1.56)	− 0.53 (− 2.65, 1.58)	− 1.43 (− 2.22, − 0.64)	− 0.32 (− 2.06, 1.41)	− 2.43 (− 5.13, 0.27)
Post-intervention linear trend**	− 0.28 (− 2.17, 1.60)	− 0.27 (− 2.39, 1.84)	− 1.28 (− 2.07, − 0.49)	− 0.13 (− 1.87, 1.60)	− 2.25 (− 4.95, 0.45)

Jan. 2015–March. 2020 relative to April. 2020–Nov. 2020.

*P-Heterogeneity for the EBF slope change coefficient.

^†^Nov. 2011–Dec. 2014 relative to Jan. 2009–Oct. 2011.

^‡^Monthly change Nov. 2011–Dec.2014.

^§^Jan. 2009–Dec. 2014 relative to Jan. 2015–Nov. 2020.

^¶^Monthly change Jan. 2015- Nov.2020.

**Monthly change April. 2020–Nov.2020.

## Discussion

Our study assessed the impact on the EBF prevalence trend at 3 and 6 months of two health policies implemented in Chile and the COVID-19 pandemic, using the national public healthcare system aggregated municipal data. Our results showed that at 3 months, the extra 12 weeks of mandatory ML did not change the EBF trend, while the P4P strategy increased EBF prevalence by 3% and COVID-19 decreased it by 5%. At 6 months, the P4P strategy increased EBF by 6%, with no changes were observed after the extra 12 weeks of ML or COVID-19. Stratified analysis by urbanicity and geographical macrozones showed an unequal effect of the two policies and COVID-19 on EBF.

Our null results of the additional 12 weeks of ML over EBF do not coincide with a previous study that found an increase of 1.8% in EBF at 6 months. However, this was a descriptive study with a shorter analysis period (2008–2013)^12^. A study performed in the United States, where there is no mandatory fully-paid ML, described a modest increase in EBF at 6 months after the implementation of voluntarily implemented paid ML^34^. A Brazilian study, where ML consist of 120 days of fully-paid ML, that included two different large surveys in 2008 and 2014, also showed an increase in EBF at 4 months and an increase in the number of women accessing paid ML. This study highlights the importance of ML access for the success of EBF^35^. In addition, a literature review reported that a longer paid ML was associated with longer EBF in all studies included in this review^8^. One possible explanation for our null results could be the low access to mandatory ML among women affiliated to the public health system, which reached only a mean of 20% during the study period^22,29^. This 20% comprises only women with formal contracts. In addition, Chilean data has already reported a high percentage of women working under informal conditions, which could be reflected in the low ML access^36^. Another explanation for the lack of effect of the mandatory ML on EBF could be that the total duration of ML in Chile is 24 weeks, which equals only five and a half months. A ML that does not reach 6 months increases the chance of an early EBF interruption by introducing formula milk or solid food before mothers return to work. On the other hand, the 24 weeks of ML allows mothers to give EBF for at least 3 months, which was not possible with the previous 12 weeks of ML (two and a half months). This shows that an eventual extension of the ML to 6 months or more could potentially reflect an effect on EBF at 6 months. Moreover, the lack of impact of the extra 12 weeks on ML could reflect a need of providing EBF strategies together with the ML, as a mandatory ML by itself might not be sufficient to promote EBF^1^.

The P4P strategy increased the national EBF prevalence by 3.1% and 5.7% at 3 and 6 months, respectively. Studies assessing other outcomes such as diabetes control, tobacco use, and drug addiction have also shown positive results with P4P strategies^9^. To the best of our knowledge, no previous published study has yet analysed the impact of P4P strategies on EBF promotion in primary healthcare settings. The increase on EBF observed after the implementation of the P4P strategy could be explained by the additional resources secured by the Ministry of Health and the Ministry of Social Welfare under the *Chile Crece Contigo* (Chile Grows with You) program^37^, which aims to promote EBF at 6 months. The added funds included activities with health professionals in hospitals and primary healthcare, as well as with mothers, partners, and their families during pre- and postnatal period, and further regulation of formula milk marketing and sales. Nonetheless, health professionals reporting EBF are the same who receive the monetary incentives, which could potentially lead to misreporting, however, our study did not aim to probe misreporting. A Chilean study that registered EBF information from mothers at the time of children’s vaccination reported a much lower EBF prevalence than the national average (32.4% at 4 months and 8.8% at 6 months), concluding that national EBF data at 6 months could be misreported^38^. Our study also reported an increase in EBF at 3 months after the implementation of the P4P strategy, where no bonuses are offered to health professionals, showing that P4P could increase EBF without being affected by the record system and bonuses. In addition, the stratified data showed the same trend as the national data after the implementation of P4P, providing evidence that the increase on EBF after the P4P is recurrent in the whole country. Furthermore, a Chilean study observed that P4P incentives can improve the performance of primary health care dental practices and that these seem to be a useful strategy to enhance oral healthcare providers performance^39^. Finally, we cannot rule out that the effect shown by the P4P strategy on EBF could be an accumulated result of a long-term interaction of the extended ML and the P4P strategy.

The COVID-19 pandemic reduced the national EBF prevalence only at 3 months. Nevertheless, these results should be interpreted with caution because of the short COVID-19 period covered in the analyses. COVID-19 restrictions have been associated with fewer healthcare appointments, an intensification of housekeeping and household care loads, and a rise in stress levels^17,40^. Studies have shown a reduction of up to 50% in health coverage as an indirect effect of COVID-19^41,42^. An Irish study found two different EBF behaviours; while some mothers interrupted EBF earlier than planned, others were able to give EBF for a longer than planned period because they stayed at home^42^. This evidence mix might explain the null impact of COVID-19 on EBF at 6 months in our study.

To the best of our knowledge, our study is unique in evaluating the impact of the addition of 12 weeks of ML, a P4P strategy in primary healthcare to promote EBF and COVID-19 on EBF by geographic zone and urban/rural residence in Chile. It has previously been reported that urbanicity is associated with lower EBF prevalence^43^, also observed in our analyses. We observed that the P4P strategy had a positive impact on EBF at 6 months only in urban areas, probably because of difficulties in reaching health services and less resource allocation for EBF promotion activities in rural areas^18^. We identified an unequal effect of the extended ML and the P4P on EBF when stratifying by macrozones and urban/rural settings, showing heterogeneity. These findings could be explained by dissimilar implementation approaches to P4P at the local level, where each municipality administers the resources provided by the Ministry of Health, showing dependency on local conditions such as geographic isolation, socio-economic status, and healthcare access. Our study reported a decrease in EBF at 3 months during COVID-19 only in the Big North. This zone has several extremely poor and isolated municipalities and a high immigration rate. In addition, during the first COVID-19 wave in Chile, the Big North had longer lockdowns than the rest of the country^44^.

The main strength of our study is the strong statistical analysis provided by ITSA. In addition, we used the nationwide public healthcare system user’s dataset, representing 80% of Chilean mothers. Furthermore, our analyses included a substantial number of data points, providing strong statistical power to our results.

Our study has several limitations. First, we did not include a comparison control group; however, our ITSA analyses incorporated the pre-intervention trend as an historical control time line or counterfactual period^32^. Second, our study did not include EBF data from the wealthiest mothers, who are registered in the private healthcare system, representing approximately 20% of the population^22^, because only the public system gathers EBF information. Therefore, our results can only be extrapolated to those affiliated to the public healthcare system or similar populations**.** Third, issues related to under-reporting or over-reporting could bias our results; nonetheless, we analysed a large nationwide dataset, which provides robustness to our findings. Finally, as previously mentioned, the COVID-19 period evaluated was short; hence, further research and analyses, incorporating a more extended period and extra data points, should be considered to provide conclusions of the full COVID-19 impact on EBF prevalence in Chile. COVID-19 has already been shown to decrease EBF, particularly in lower-income population^13^.

## Conclusion

No effect was observed on EBF by the addition of 12 weeks of mandatory ML. The P4P strategy increased EBF at 3 months by 3.1% and 5.7% at 6 months. The COVID-19 pandemic decreased EBF at 3 months by 5%. These results suggest that the current five and a half months of ML and the low access to ML among those affiliated to the public health sector (only 20%) limit the impact of the extra 12 weeks of ML on EBF. Our results also suggest that the P4P is a successful strategy for promoting and increasing EBF across the country. However, improved national and regional uniformity in P4P resource distribution could counterbalance the regional differential impact identified on EBF. Extra monitoring and record validation should be implemented to ensure reliable data, minimizing under-reporting or over-reporting. To reach the WHO goal of 70% EBF by 2030^5^, the Chilean government should consider increasing accessibility and ML extension and accessibility, as well as implementing permanent support for multi-level actions such as the P4P strategy. EBF promotion and healthcare-related activities should be maintained and protected during emergencies (e.g., pandemics).

## Data Availability

The data that support the findings of this study are available from the Chilean Ministry of Health and the Maternity Leave Management and Information System [SIMAT], from the Social Welfare Superintendence (SUSESO)^29^. Restrictions apply to the availability of these data, which was used under license for the current study; therefore, these data are not publicly available. Data are, however, available from the authors (D.N.R and M-L.G) upon reasonable request and with permission of the Chilean Ministry of Health and the Maternity Leave Management and the Social Welfare Superintendence (SUSESO).
